# Case fatality of agricultural pesticides after self-poisoning in Sri Lanka: a prospective cohort study

**DOI:** 10.1016/S2214-109X(21)00086-3

**Published:** 2021-04-23

**Authors:** Nicholas A Buckley, Mohamed Fahim, Jacques Raubenheimer, Indika B Gawarammana, Michael Eddleston, Michael S Roberts, Andrew H Dawson

**Affiliations:** a South Asian Clinical Toxicology Research Collaboration, Faculty of Medicine, University of Peradeniya, Kandy, Sri Lanka; b Faculty of Allied Health Sciences, Department of Pharmacy, University of Peradeniya, Kandy, Sri Lanka; c Pharmacology, Biomedical Informatics and Digital Health, Faculty of Medicine and Health, University of Sydney, Sydney, NSW, Australia; d Centre for Pesticide Suicide Prevention, and Pharmacology, Toxicology and Therapeutics, Centre for Cardiovascular Science, University of Edinburgh, Edinburgh, UK; e Basil Hetzel Institute for Translational Health Research, UniSA Clinical and Health Sciences, University of South Australia, Adelaide, SA, Australia; f Diamantina Institute, University of Queensland, Brisbane, QLD, Australia

## Abstract

**Background:**

Pesticide poisoning is among the most common means of suicide globally, but can be prevented with regulation of the most hazardous agents. We aimed to compare the lethality of pesticides ingested by our cohort, seek evidence on variation between human and regulatory animal toxicity, and establish change over time in the case fatality of individual pesticides in Sri Lanka.

**Methods:**

We examined the case fatality of agricultural pesticides in a prospective cohort in nine hospitals serving rural populations in Sri Lanka. We included all patients (>11 years) who had presented to a South Asian Clinical Toxicology Research Collaboration study hospital during the study period. Patients were enrolled by clinical research assistants and were regularly reviewed. Identification of the ingested pesticide was generally on the basis of history or positive identification of the container, supported by nested blood analysis.

**Findings:**

From March 31, 2002, to Dec 31, 2019, 34 902 patients (median age 29 years [IQR 21–40]; 23 060 [66·1%] male) presented with a possible or known pesticide self-poisoning. We identified 23 139 specific pesticides that were ingested. Poisoning was fatal in 2299 (6·6%) patients. Case fatality varied greatly from 0·0% (several substances) to 41·8% (paraquat). The three most toxic agents (ie, paraquat, dimethoate, and fenthion) were banned between 2008 and 2011. Since 2013, the five agents causing the most deaths (ie, profenofos, propanil, fenobucarb, carbosulfan, and quinalphos) had a case fatality of 7·2–8·6%. A steady decline was seen in overall case fatality of pesticide poisoning (10·5% for 2002–06 to 3·7% for 2013–19), largely attributable to pesticide bans. A modest fall in case fatality for non-banned pesticides was also seen.

**Interpretation:**

Declines seen in case fatalities of poisonings with non-banned pesticides suggest that medical management improved over time. The human data for acute toxicity of pesticides should drive hazard classifications and regulation. We believe that a global benchmark for registration of pesticides should include a less than 5% case fatality after self-poisoning, which could prevent many deaths and have a substantial effect on global suicide rates.

**Funding:**

The Wellcome Trust and the National Health and Medical Research Council of Australia.

**Translations:**

For the Sinhala and Tamil translations of the abstract see Supplementary Materials section.

## Introduction

WHO has reported that pesticide self-poisoning is one of the most common means of suicide globally. It is also the most preventable method of suicide, through better pesticide regulation.^1^ Despite a fall in deaths due to pesticide poisonings in the past two decades from around 260 000 a year to 160 000 a year, each year more than 150 000 people die from deliberate ingestion of pesticides accounting for about 20% of the global burden of suicide.^2^ Falls in deaths are believed to be due to tighter regulation and increased mechanisation of agriculture resulting in reduced numbers of agricultural workers. In many developing countries in the Asia-Pacific region, suicide is the leading cause of death in early to middle adult life,^3^ and pesticides in this region account for around a half to two-thirds of suicides.

A highly effective strategy to reduce mortality is to restrict access to the more toxic pesticides.^4^, ^5^ Restriction of agricultural use of acutely toxic, highly hazardous pesticides has repeatedly been shown to reduce overall poisoning and suicide deaths, in Sri Lanka,^6^ South Korea,^7^ Bangladesh,^8^ and elsewhere.^5^, ^9^ These restrictions have had no measurable adverse effect on agricultural productivity.^6^, ^7^, ^8^, ^9^ We have previously shown that acute lethal human toxicity is poorly predicted by animal studies, and we argued for regulatory strategies to prioritise focus on human data.^4^, ^5^ The optimal regulatory strategy should be based around human morbidity and mortality and adapted to each country's agricultural need and documented poisoning issues.^5^, ^9^

We established a prospective cohort of patients with intentional pesticide self-poisoning in Sri Lankan hospitals in 2002, to study the acute toxicity of pesticides in humans after ingestion of agricultural formulations. Many changes have been made in the health system in Sri Lanka over the past two decades that might alter the risk of death. These changes include more emergency departments, post-graduate training in toxicology, wider availability of antidotes, better evidence to guide management, and updated guidelines.^10^ There were also bans of three highly hazardous pesticides between 2008 and 2011 (ie, dimethoate, fenthion, and paraquat) and bans of four common pesticides from 2013 to 2016 for reasons unrelated to acute toxicity (ie, chlorpyrifos, glyphosate, carbosulfan, and carbaryl).^11^ In this study, we aimed to compare the lethality of all pesticides ingested by our cohort, seek more evidence on the variation between human and regulatory animal toxicity, and establish whether there has been any change over time in the case fatality of individual pesticides in acute human self-poisoning in Sri Lanka.

**Research in context**

**Evidence before this study**

No other large, prospective, cohort studies have been done for pesticide poisoning. Bans of highly hazardous pesticides reduce deliberate and accidental fatal poisonings. Current WHO classifications of pesticide hazards are largely informed by animal median lethal doses. This method ignores species differences, the formulations used, and responsiveness to medical treatment. The case fatality of human pesticide poisonings can differ greatly from that predicted by the WHO classification.

**Added value of this study**

The case fatality of agricultural pesticides in our prospective cohort of pesticide self-poisonings in Sri Lanka showed very large variation in case fatality for agents with the same WHO hazard classifications. We also showed a reduction in overall case fatality after bans of the three most toxic agents between 2008 and 2011. We highlight the higher case fatality of five common pesticides (all WHO class II, moderately hazardous) disproportionately responsible for deaths currently (ie, profenofos, propanil, carbosulfan, fenobucarb, quinalphos), and provide data establishing the lower acute human toxicity of dozens of other safer pesticides.

**Implications of all the available evidence**

If human data for acute toxicity of pesticides was used for hazard classifications and regulation worldwide, it would prevent many deaths and have a substantial effect on global suicide rates. A further 10–15% reduction in global suicide rates would be achievable with coordinated global action.

## Methods

### Study design and participants

We did a prospective, observational, cohort study of all patients older than 11 years with deliberate ingestion of pesticides who presented with poisoning to nine Sri Lankan referral hospitals predominantly serving surrounding rural populations, located in four provinces in Sri Lanka (central, northwestern, north-central, and southern). The type of poison ingested was established from the history given by the patient or relatives, the hospital transfer letter, from the pesticide bottle if it accompanied the patient, or occasionally from toxicological analysis. Patients were either direct admissions to the study hospital or transfers from smaller primary hospitals. Patient recruitment commenced on March 31, 2002, with varying periods covered for the nine hospitals since that time. Within this cohort there have been, over time, various other research questions requiring the recruitment of particular patients, thus influencing which hospitals were study hospitals at different points over the study period. Initial large studies were clinical trials of antidotes.^12^, ^13^ In the past 8 years, studies have been of neurological and renal complications^14^, ^15^ and education and community interventions.^16^ For our study, data analysis was done on all patients who had presented to a South Asian Clinical Toxicology Research Collaboration study hospital up until Dec 31, 2019—including those patients reported in an earlier overall cohort analysis.^4^ Recruitment decreased substantially after 2016 due to only one hospital continuing recruitment because of decreased research funding. Therefore, when showing yearly changes in case fatality, 2017–19 was taken as one time unit.

Patients were enrolled into the cohort by clinical research assistants. Clinical care was determined by the treating physicians but generally followed standard protocols that were driven by the correct diagnosis of the poison ingested. Patients were regularly reviewed and clinically significant complications (eg, intubation, seizures, or death) recorded prospectively.

Ethical approval for this data collection was obtained from the research ethics committees of Colombo University Faculty of Medicine; Sri Lankan Medical Association; University of Peradeniya Faculty of Medicine; Australian National University; University of New South Wales; University of Sydney; Oxfordshire Clinical Research Ethics Committee; and Oxford Tropical Medicine Ethics Committee. The need for informed consent was waived for observational cohort data collection, but many patients provided written informed consent to be enrolled in specific studies.

### Procedures

We previously analysed blood samples from patients with known poisoning, finding that laboratory analysis confirmed the history in 95% of cases.^17^, ^18^ However, our cohort included 15–20% of patients who had ingested an unknown pesticide (or unknown organophosphorus insecticides, among others), with a high case fatality. There were also many poisonings caused by unknown substances, which might also be pesticides. We attempted to identify the agents involved in 664 poisoning cases, focusing on unknown poisonings that were severe enough to cause a coma or death. 1353 samples had been collected from these 664 patients on admission (typically 4–5 h after ingestion). These samples were assayed for pesticide concentration in the Basil Hetzel Institute for Translational Health Research (Adelaide, SA, Australia). The assays were by liquid chromatography or tandem mass spectrometry, as published.^19^, ^20^ 31 pesticides were covered by the assays, selected from those that we had developed assays for in previous studies or present in pesticide libraries.

We used the WHO Recommended Classification of Pesticides by Hazard (Ia [extremely hazardous], Ib [highly hazardous], II [moderately hazardous], III [slightly hazardous], and U [unlikely to present acute hazard]).

### Statistical analysis

Case fatality was defined as the proportion of fatal outcomes after poisoning admission for any given pesticide. Clopper-Pearson exact 95% CIs of the proportions were calculated. This calculation was done annually for all pesticides, for each agent, and, for the nine most common agents and three most common unknown categories, across three time periods (2002–06 [pre-bans], 2007–12 [during the time when paraquat, fenthion, and dimethoate were banned to reduce poisoning deaths], and 2013–19 [after these bans, when glyphosate, carbofuran, and chlorpyrifos were banned in 2015 due to concerns about chronic effects not acute toxicity; appendix 3 p 1).^11^, ^21^ Note that case fatality was calculated for each substance, so that the small numbers of patients who had ingested multiple substances were counted for each substance in question.

Case fatality varies with age and sex, and has fallen over time, perhaps partly due to improving medical resources and training. We did logistic regression using PROC LOGISTIC in SAS, version 9.4, and calculated Wald 95% CIs on the odds ratios. The first analysis aimed to establish if factors other than specific pesticide were confounding the relative toxicity of agents. We used the pesticides as the classification variable with chlorpyrifos as the reference group, modelling the event of death as the outcome, adjusting for age, sex, and year. The second analysis aimed to determine the effect of banning the three more toxic agents (ie, dimethoate, fenthion, and paraquat) on the overall case fatality, compared with other factors. The proportion of known pesticide poisonings due to these three agents each year, age, sex, hospital, and year were included in this model.

### Role of the funding source

The funders of the study had no role in study design, data collection, data analysis, data interpretation, or writing of the report.

## Results

**Figure 1:**
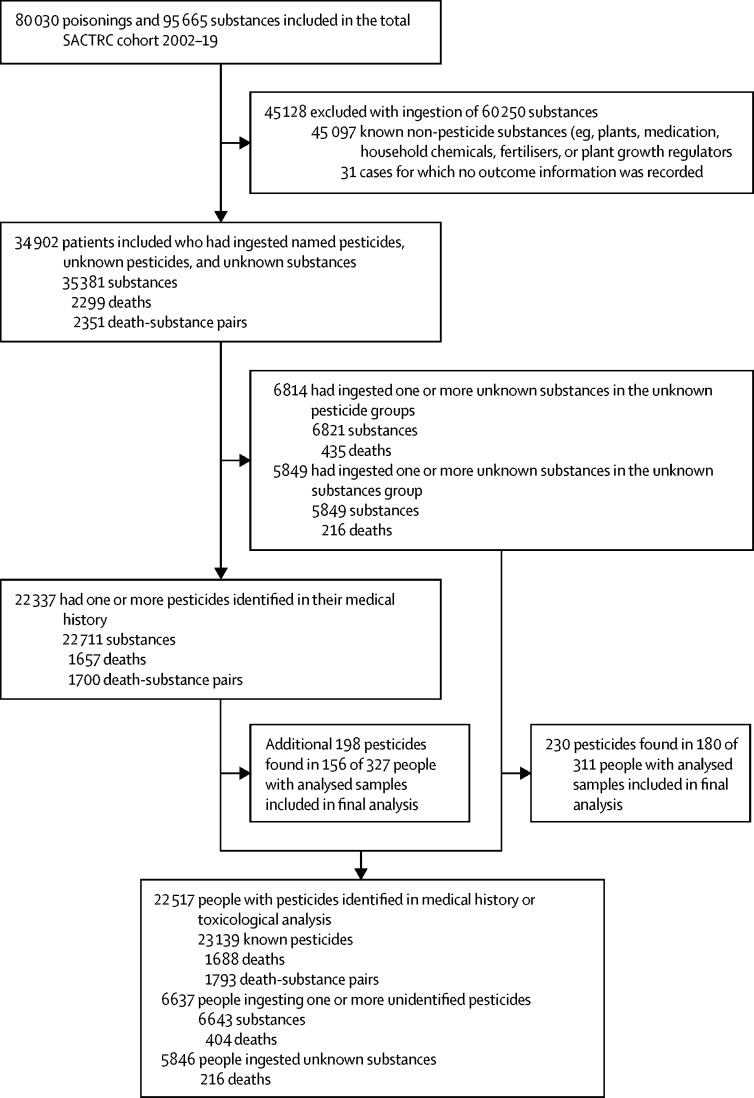
Study profile Death-substance pairs are higher than the total number of deaths as deaths were counted against each pesticide ingested. In the lower half of the figure, people and deaths add up to more than 34 902 and 2299 as there were 98 people (nine deaths) who ingested known pesticides and other unknown pesticides or substances and are counted twice. SACTRC=South Asian Clinical Toxicology Research Collaboration.

From March 31, 2002, to Dec 31, 2019, 80 030 patients were enrolled in the South Asian Clinical Toxicology Research Collaboration cohort. Figure 1 shows the process of delineating the final study sample. We excluded 45 097 ingestions due entirely to known non-pesticide substances (eg, plants, medication, household chemicals, fertilisers, or plant growth regulators) and 31 cases for which no outcome information was recorded. 34 902 patients ingested 35 381 pesticides or unknown substances that were possibly pesticides (including cases where a pesticide was suspected and ignoring non-pesticide co-ingestions); 455 patients took more than one pesticide. This cohort includes the 9302 patients reported previously.^4^ We refer to patients, but it is probable that a few people appear more than once in our cohort. The Sri Lankan hospital medical records are separate for each episode of care. In contrast to high-income countries, repeated self-poisoning is uncommon and carries the same risk on each occasion.^22^

Across the cohort, 638 patients had blood samples analysed for the pesticide responsible for the poisoning; 230 new pesticides causing patient poisonings were identified in 180 cases of poisoning with unknown pesticides (n=226) or unknown substances (n=4); an additional 198 pesticides were found in 180 people who already had known pesticides identified from the patient's history (figure 1).

2299 patient deaths were recorded (for 2351 substance–death pairs). Deaths were recorded against 3·7% (216 of 5846) of the unknown substance poisonings, 6·1% (404 of 6643) of the unknown pesticide poisonings, and 7·7% (1793 of 23 139) of all the known pesticide poisonings.

Most individuals in the sample were male (23 060 [66·1%] of 34 902), with a median age of 29 years (IQR 21–40). Most patients consumed only one poison (28 552 [81·8%]), although this number included all patients for whom the ingested substance was unknown (and could thus potentially have been multiple substances), and the most recorded substances ingested was six (n=1). Two-thirds (23 245 [66·6%]) of patients were transferred from smaller peripheral hospitals to the study hospital, and 2299 (6·6%) patients died. The time since ingestion was either directly recorded (n=16 795) or could be inferred from other values in 32 114 patients. The median time from ingestion was 225 min (IQR 130–405). These variables changed minimally over time (appendix 3 p 2).

**Figure 2:**
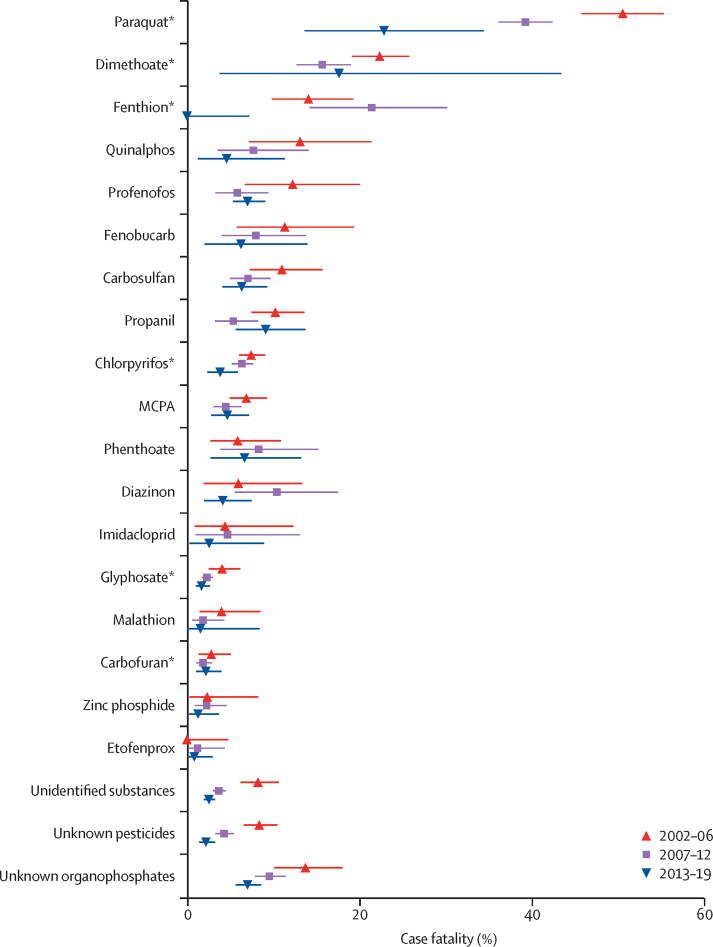
Changes over time in case fatality for the 18 most common pesticides and three largest unknown categories for 2002–06 versus 2007–12 versus 2013–19 Data are case fatality (95% CI). *Pesticides banned between 2010 and 2015.

**Figure 3:**
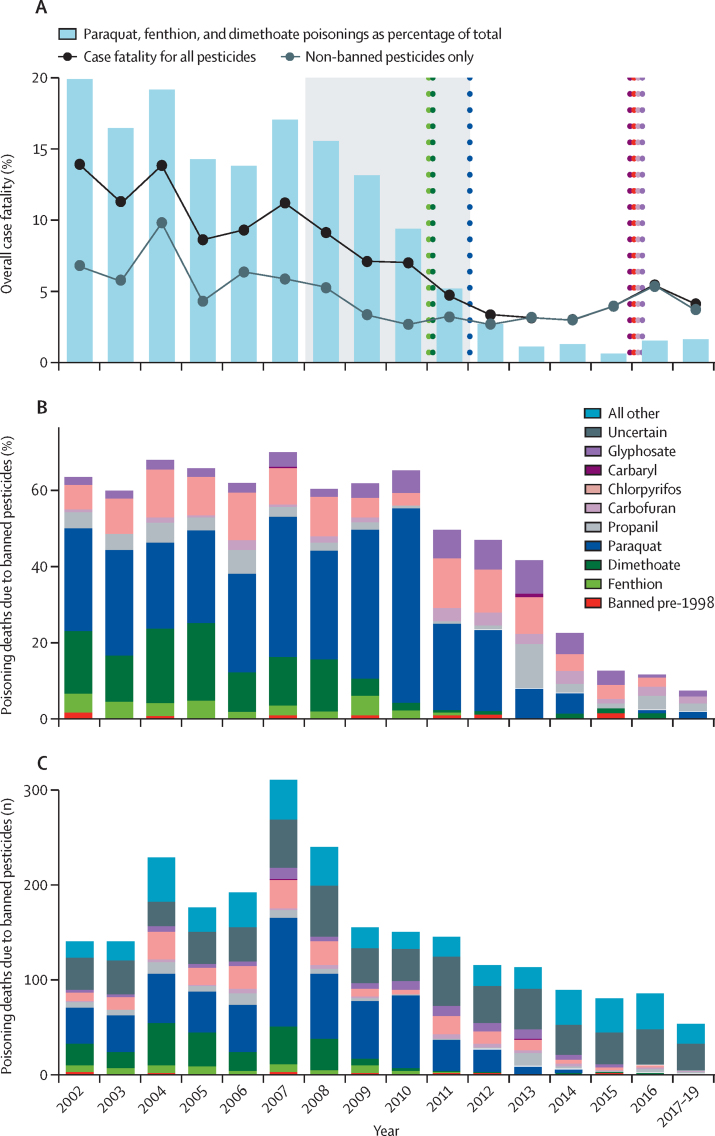
Pesticide bans and changes in case fatalities, 2002–19 Timing of seven bans and changes in overall pesticide case fatality (A), percentage of poisoning deaths due to banned pesticides (B), and the number of poisoning deaths due to banned pesticides (C). In A, the grey shaded area shows years when there were progressive restrictions on imports. The four later bans shown were for environmental reasons. Although the number of poisonings from these agents was reduced, the bans did not contribute to reduced case fatality. Propanil is listed as it was subject to a regional but not national ban from 2015.

The case fatality for all pesticides is presented in the table, and the changes over time in the case fatality of individual agents are shown in figure 2. For most agents, case fatality remained similar or fell slightly over time; however, more marked reductions were seen for paraquat (figure 2). The overall annual case fatality for pesticide self-poisoning (including patients who had ingested an unidentified pesticide) more than halved over 18 years (figure 3A). Anticholinesterase (organophosphorus and carbamate) insecticides and the herbicides paraquat, MCPA, propanil, and glyphosate accounted for 19 295 (83·4%) of 23 139 admissions and 1727 (96·3%) of 1793 deaths for which specific agents were identified. The case fatality was generally much higher for those agents that have now been banned (table, appendix 3 p 3). The bans were highly effective at reducing poisonings with banned agents, although there were 59 poisonings from banned pesticides over the 18 years (figure 3). There remain five commonly ingested agents (ie, profenofos, carbosulfan, propanil, quinalphos, and fenobucarb; table, figure 2) that have greater toxicity than any of the agents that have been banned from 2013 to 2016 (ie, chlorpyrifos, carbaryl, carbofuran, and glyphosate), and these are now the most commonly identified agents in fatal poisonings. These five agents have caused 100 (23·9%) of 419 deaths since 2013, while being only 12·8% of all pesticide poisonings. They are also likely to be over-represented in the 174 unknown fatal pesticide poisonings in this time.

**Table:** Case fatality of any pesticide associated with ten or more admissions

		**Number of patients**	**Deaths**	**Case fatality (95% CI)**	**WHO toxicity class**
Organophosphorus insecticides		10 612	927	8·7% (8·2–9·3)	
	Dimethoate*	1190	229	19·2% (17·0–21·6)	II
	Fenthion*	388	56	14·4% (11·1–18·3)	II
	Prothiofos	22	3	13·6% (2·9–34·9)	II
	Quinalphos	303	26	8·6% (5·7–12·3)	II
	Methamidophos*	35	3	8·6% (1·8–23·1)	Ib
	Azinphos-methyl*	12	1	8·3% (0·2–38·5)	Ib
	Profenofos	1161	84	7·2% (5·8–8·9)	II
	Phoxim	14	1	7·1% (0·2–33·9)	II
	Phenthoate	366	25	6·8% (4·5–9·9)	II
	Chlorpyrifos*	3340	213	6·4% (5·6–7·3)	II
	Diazinon	448	27	6·0% (4·0–8·6)	II
	Monocrotophos*	29	1	3·4% (0·1–17·8)	Ib
	Malathion	482	12	2·5% (1·3–4·3)	III
	Acephate	51	1	2·0% (0·0–10·4)	II
	Pirimiphos-methyl	20	0	0·0% (0·0–16·8)	II
	Azamethiphos	25	0	0·0% (0·0–13·7)	II
	Coumaphos*	35	0	0·0% (0·0–10·0)	Ib
	Other organophosphorus insecticides	51	11	21·6% (11·3–35·3)	NA
	Unknown organophosphorus insecticides	2640	234	8·9% (7·8–10·0)	NA
Carbamate insecticides		3440	165	4·8% (4·1–5·6)	
	Fenobucarb	315	27	8·6% (5·7–12·2)	II
	Carbosulfan	1129	86	7·6% (6·1–9·3)	II
	Carbaryl*	30	2	6·7% (0·8–22·1)	II
	Methomyl*	26	1	3·8% (0·1–19·6)	Ib
	Carbofuran*	1740	37	2·1% (1·5–2·9)	Ib
	Propoxur	68	1	1·5% (0·0–7·9)	II
	Other carbamates	7	0	0·0% (0·0–41·0)	NA
	Unknown carbamates	125	11	8·8% (4·5–15·2)	NA
Pyrethroids		1046	5	0·5% (0·2–1·1)	
	Fenvalerate	11	1	9·1% (0·2–41·3)	II
	Etofenprox	482	4	0·8% (0·2–2·1)	U
	Cyfluthrin	12	0	0·0% (0·0–26·5)	Ib
	Imiprothrin	14	0	0·0% (0·0–23·2)	II
	λ-cyhalothrin	16	0	0·0% (0·0–20·6)	II
	Deltamethrin	32	0	0·0% (0·0–10·9)	II
	Cypermethrin	42	0	0·0% (0·0–8·4)	II
	Flumethrin	51	0	0·0% (0·0–7·0)	NA
	Allethrin	85	0	0·0% (0·0–4·2)	II
Other pyrethroids		41	0	0·0% (0·0–8·6)	NA
	Unknown pyrethroids	260	0	0·0% (0·0–1·4)	NA
	Other insecticides	1008	20	2·0% (1·2–3·0)	
	Endosulfan*	15	3	20·0% (4·3–48·1)	II
	Chlorantraniliprole	17	1	5·9% (0·1–28·7)	U
	Imidacloprid	218	8	3·7% (1·6–7·1)	II
	Tebufenozide	29	1	3·4% (0·1–17·8)	U
	Chlorfluazuron	110	2	1·8% (0·2–6·4)	U
	Thiamethoxam	56	1	1·8% (0·0–9·6)	II
	Abamectin	254	4	1·6% (0·4–4·0)	Ib
	Spinosad	10	0	0·0% (0·0–30·8)	III
	Azadirachtin	11	0	0·0% (0·0–28·5)	NA
	Acetamiprid	42	0	0·0% (0·0–8·4)	II
	Fipronil	122	0	0·0% (0·0–3·0)	II
	Other	124	0	0·0% (0·0–2·9)	NA
Herbicides		9054	904	10·0% (9·4–10·6)	
	Paraquat*	1477	618	41·8% (39·3–44·4)	II
	Quinclorac	24	3	12·5% (2·7–32·4)	III
	Propanil	982	81	8·2% (6·6–10·1)	II
	Clomazone	26	2	7·7% (0·9–25·1)	II
	Alachlor*	13	1	7·7% (0·2–36·0)	II
	Glufosinate-ammonium	48	3	6·3% (1·3–17·2)	II
	Thiodicarb	18	1	5·6% (0·1–27·3)	II
	MCPA	1646	88	5·3% (4·3–6·5)	II
	Bispyribac-sodium	227	7	3·1% (1·2–6·3)	III
	Glyphosate*	3908	93	2·4% (1·9–2·9)	III
	Pyribenzoxim	59	1	1·7% (0·0–9·1)	NA
	Pretilachlor	119	2	1·7% (0·2–5·9)	U
	Fenoxaprop-P-ethyl	173	1	0·6% (0·0–3·2)	III
	Benthiocarb	11	0	0·0% (0·0–28·5)	II
	Diuron	14	0	0·0% (0·0–23·2)	III
	Dithiocarbamate	19	0	0·0% (0·0–17·6)	NA
	Pendimethalin	20	0	0·0% (0·0–16·8)	II
	Ethephon	22	0	0·0% (0·0–15·4)	III
	Oxyfluorfen	34	0	0·0% (0·0–10·3)	U
	Other herbicides	75	1	1·3% (0·0–7·2)	NA
	Unknown herbicides	139	2	1·4% (0·2–5·1)	NA
Fungicides		352	6	1·7% (0·6–3·7)	
	Edifenphos†	19	2	10·5% (1·3–33·1)	Ib
	Hexaconazole	28	1	3·6% (0·1–18·3)	III
	Carbendazim	19	0	0·0% (0·0–17·6)	U
	Copper oxide	23	0	0·0% (0·0–14·8)	II
	Propineb	32	0	0·0% (0·0–10·9)	U
	Tebuconazole	32	0	0·0% (0·0–10·9)	II
	Mancozeb	36	0	0·0% (0·0–9·7)	U
	Chlorothalonil	65	0	0·0% (0·0–5·5)	U
	Other fungicides	73	3	4·1% (0·9–11·5)	NA
	Unknown fungicides	25	0	0·0% (0·0–13·7)	NA
Rodenticides		859	12	1·4% (0·7–2·4)	
	Zinc phosphide	635	12	1·9% (1·0–3·3)	Ib
	Ethanethiol	10	0	0·0% (0·0–30·8)	NA
	Brodifacoum	36	0	0·0% (0·0–9·7)	Ia
	Coumarin	39	0	0·0% (0·0–9·0)	Ib
	Bromadiolone	98	0	0·0% (0·0–3·7)	Ia
	Other rodenticides	12	0	0·0% (0·0–26·5)	NA
	Unknown rodenticides	29	0	0·0% (0·0–11·9)	NA
Miscellaneous					
	Other pesticides	10	1	10·0% (0·3–44·5)	NA
	Unknown pesticides	3402	157	4·6% (3·9–5·4)	NA
	Unidentified substances	5845	216	3·7% (3·2–4·2)	NA

Data for all substances is shown in the appendix 3 (p 3). NA=not applicable. The WHO Recommended Classification of Pesticides by Hazard ranges from Ia (extremely hazardous), Ib (highly hazardous), II (moderately hazardous), III (slightly hazardous), U (unlikely to present acute hazard), and O (obsolete as pesticide, not classified).^23^

* Substances are now banned in Sri Lanka.

† Also has anticholinesterase activity.

There was a very wide range of case fatality for compounds within the same pesticide classes (eg, anticholinesterase insecticides: 2·5% for malathion *vs* 19·2% for dimethoate) or with the same agricultural indication (eg, herbicides: 0·6% for fenoxaprop-P-ethyl *vs* 41·8% for paraquat), although the variation in agents that have not been banned was much less (table). There was a modest relationship between case fatality and WHO classification of hazard.^23^ A fatal outcome was more likely to occur with increasing age, male sex, and earlier years. However, adjustment for these factors did not have any substantial effect on the odds ratios for the relative risk of death from common pesticide poisonings (appendix 3 p 9). Logistic regression showed the proportion of pesticides due to the banned agents explained much of the change over the years (appendix 3 p 10). The odds of fatal poisoning increased 1·055 times for each percentage of annual poisonings involving these agents. The approximate 16% reduction in these agents therefore explained a 50–60% reduction in overall case fatality.

Incorporating the laboratory results increased the known pesticide substance count from 22 711 to 23 139, but these small numbers had little effect on estimates of case fatality. However, we identified a small number of poisonings with WHO hazard class I or obsolete organophosphorus pesticides,^23^ which had been banned or never registered in Sri Lanka during this time—azinphos-methyl (n=12), parathion-methyl (n=7), carbophenothion (n=2), chlorfenvinphos (n=2), bromophos-ethyl (n=2), and ethion (n=1).

## Discussion

This study provides evidence that falls in pesticide poisoning deaths in Sri Lanka^6^ are attributable to effective implementation of bans of highly hazardous pesticides. We also provide further evidence of continuing large differences in human case fatality between pesticides that are used for similar agricultural indications. The findings highlight that some of the most common pesticides remaining in agricultural use in Sri Lanka (eg, profenofos, carbosulfan, fenobucarb, quinalphos, and propanil) have higher than average human toxicity. The higher toxicity of these specific pesticides is not apparent from the WHO class II rating based on animal median lethal dose (LD_50_) values, as there are many much less lethal agents in this class (eg, fipronil and allethrin). Further, there were many pesticides across all categories and indications that had a demonstrable case fatality of less than 5%. This result would be a currently achievable benchmark to set for a pesticide formulation to establish categorisation as highly or extremely hazardous (WHO class I), or as a highly hazardous pesticide as defined by the Food and Agriculture Organization of the United Nations and WHO,^23^, ^24^ with the resulting regulatory consequences. The improved outcome for most individual pesticide poisonings in Sri Lanka over time suggests medical management is improving within our cohort. There could be many areas of the rural developing world that are less well resourced where these more hazardous pesticides would have a much higher case fatality. Further targeted bans of these agents would probably lead to further modest reductions in fatal poisonings and suicides. Profenofos, in particular, also very commonly causes protracted respiratory failure resulting in high morbidity.^15^

Before its complete ban, the case fatality for paraquat poisoning fell markedly (from 50% to 23%; figure 2), which was likely to be due to two formulation changes. First, Syngenta (Basel, Switzerland) introduced a 20% formulation designed specifically to reduce absorption after ingestion, followed by a reduction in all paraquat product maximum permitted concentrations from 20·0% to 6·5%.^11^ Although neither change was sufficient to reduce case fatality to match that of other herbicides, the reductions do highlight the considerable potential for changed formulations to impact case fatality.

We also noted the occasional fatal poisoning due to agents that have been banned, suggesting there is some circumvention of restrictions. Pesticide assays confirmed the presence of some of these agents, indicating that the substances had not been misidentified. Tighter enforcement of restrictions could also save lives. However, Sri Lanka's island identity has probably allowed it to more easily ban pesticides than other countries with contiguous borders with countries where bans have not taken place—eg, Nepal banned monocrotophos and parathion-methyl in 2006 but still has many cases due to illegal importation across the border with India.^25^

The striking success of pesticide bans in reducing the overall case fatality of pesticide poisoning is clearly seen in our data. This result has been mirrored in nationwide mortality data for Sri Lanka, which has now seen a 70% reduction in suicides and an even greater decline in fatal self-poisoning over two decades.^6^ Several other Asian countries (eg, Bangladesh, China, and South Korea) have also had remarkable reductions in fatal pesticide poisoning coinciding with bans of more toxic agents.^5^, ^7^, ^8^, ^9^ Targeted pesticide restrictions do not have apparent adverse effects on agricultural costs or output.^6^, ^9^

Human case fatality data are the most relevant data to guide pesticide restrictions for countries where suicide by pesticide poisoning is common. Animal data used in WHO or environmental protection agency classifications of toxicity have previously been used to guide regulation, but do not account for factors that might modify the risk, such as the toxicity of solvents, responsiveness to antidotes and supportive care, or species-specific susceptibility. Coordinated pesticide regulation of all pesticides with a high case fatality across Asia could reduce fatal pesticide poisoning by more than half and reduce total suicides in the region by at least 30%.^9^

The Food and Agriculture Organization of the United Nations and WHO Joint Meeting on Pesticide Management has developed criteria to identify the highly hazardous pesticides that cause most health and environmental harms worldwide.^24^ Criteria include acute toxicity (WHO hazard classes Ia and Ib) plus mutagenicity, carcinogenicity, and reproductive toxicity, as well as listing by the Stockholm, Rotterdam, or Montreal conventions.^24^ A final eighth criterion includes all pesticide active ingredients and formulations associated with severe or irreversible adverse effects on human health or the environment. New criteria have been proposed to include chronic risks to human health and the environment, including risks to aquatic life, terrestrial wildlife, and pollinators.^26^ However, there is no criterion for hazards after ingestion despite this factor being the cause of most acute deaths worldwide from pesticides. A criterion explicitly linked to a case fatality of more than 5%, or in time 2%, after ingestion would allow problematic WHO class II pesticides such as dimethoate, fenthion, and paraquat to be banned worldwide, as has been done so successfully in Sri Lanka. Bans of such pesticides have the potential to rapidly and substantially reduce the number of deaths from pesticide poisoning. WHO now recognises pesticide regulation as one of the most cost-effective approaches possible for suicide prevention in countries where pesticide suicides are common.^27^

The clinical and mechanistic explanations for the variation in lethality within classes and the discordance with animal toxicity data are complex and not completely understood. Four of the agents about which our data raise new concerns are anticholinesterases, with rodent LD_50_ greater than 100 mg/kg (making them only moderately hazardous in the WHO classification). Otherwise, they have little obviously in common with one another except their greater than average human toxicity. The clinical effects provide an explanation for their higher lethality. Profenofos and quinalphos are more likely to cause protracted respiratory failure than other anticholinesterases still in use in Sri Lanka,^15^ and carbamates such as fenobucarb and carbosulfan lead to a rapid onset of a severe cholinergic syndrome.^20^ From 2013 to 2019, the higher case fatality for propanil poisoning, which is easily treated, suggest that renewed efforts to educate doctors on the prompt and correct use of antidotes might be needed, as many of these deaths are probably preventable with better medical care.^28^

The key strengths of our study are that it includes a very large cohort with data collected prospectively on all acute pesticide poisonings presenting to the study hospitals during a period of 18 years. The most important limitation of our data is that it was collected in just one country. However, outcomes in these nine hospitals are likely to be representative of outcomes from rural hospitals in many low-income and middle-income countries with limited resources. Doing the study in just Sri Lanka restricted the range of pesticides for which data could be provided as many pesticides (all WHO hazard class I pesticides and endosulfan) had already been banned at the time. It would also be useful to confirm the generalisability of our findings in other cohorts. However, there are no other comparable large prospective cohort studies with careful identification of pesticides reporting case fatality for most of these agents after oral ingestion. Many of the larger studies combine deliberate self-poisoning and accidental poisonings and involve retrospective review of central records, not hospital admissions. For example, a study published in 2019 of 30 789 pesticide poisonings reported only an 11% case fatality for paraquat poisoning,^29^ which casts doubt on their other case fatality estimates as most series report a case fatality of around 50%. This result is probably due to the fact that the data were routinely sent to the state's Occupational Disease and Occupational Health Information Monitoring System, rather than being collected at the bedside by full-time clinical researchers. Our cohort's estimates are consistent with the case fatality and 95% CI estimates arising from other cohorts reporting some of the same agents we have studied.^30^

The study hospitals were secondary referral hospitals; deaths at home, in primary hospitals, and during transfers would have reduced the observed case fatality for agents that cause very rapid toxicity. However, the majority of deaths (70–90%) from pesticide poisoning occur in the secondary referral hospitals.^31^ Inclusion of patients who were well and not transferred would reduce the case fatality estimates; however, in Sri Lanka, around 80–90% of pesticide poisonings presenting to small hospitals are rapidly transferred, so non-transferred cases would largely be an issue for pesticides recognised to have very low toxicity and in any case have only a very small effect on case fatality estimates for the more toxic agents.^31^ Most of these pesticides do not cause death very rapidly, with typical times to death in hospital of more than 24 h.^14^, ^15^, ^18^ The referral hospitals included changed over time, and only one hospital recruited patients after 2017. Hospital had only a slight influence on case fatality after adjustment for other factors (appendix 3 p 10). The referral hospitals included reflected the ongoing funded studies on antidotes and toxicity. The antidote studies were negative and did not lead to new treatments being offered at later dates; however, they might have contributed to better medical management over time. We chose to examine case fatality by agent, rather than exclude the small number (1·3%) of mixed ingestions. Some pesticides are commonly sold as mixtures or have synergistic toxicity. However, counting deaths against each agent ingested in mixed ingestions might lead to the case fatality of low toxicity agents being slightly overestimated.

In conclusion, human data for acute toxicity of pesticides should drive hazard classifications and regulation. Our data further support moves to completely eliminate pesticides that have a case fatality greater than 5%, and we identify several pesticides that could be banned to reduce morbidity and mortality from pesticide poisoning in Sri Lanka. There were many serious and fatal unknown pesticide and unknown substance poisonings. Toxicological analysis identified uncommon pesticides that caused severe poisoning as well as occasional circumvention of bans and should, in theory, provide more accurate estimates of case fatality, although in our study they were not required to identify the more toxic agents. A global strategy that reclassified all the more toxic class II agents as highly hazardous and region-wide bans would prevent most circumvention and be a highly effective means of reducing suicide rates throughout the Asia-Pacific region.

## Data sharing

Individual patient data will not be made publicly available. We can provide the data dictionary for this study to anyone on request to the corresponding author. Individual de-identified participant data as described in this paper could be shared subject to governance and ethical approvals in Sri Lanka that ensure the data are only used in a manner consistent with the conditions and research purposes in the protocols under which the data was originally collected.

## Declaration of interests

ME is a WHO member of the FAO–WHO Joint Meeting on Pesticide Management, and reports receiving an unrestricted research grant from Cheminova and travel expenses from Syngenta to attend meetings. ME is affiliated with the Centre for Pesticide Suicide Prevention, which is funded by an Incubator Grant from the Open Philanthropy Project Fund, an advised fund of Silicon Valley Community Foundation, on the recommendation of GiveWell, USA. ME declares relevant grants from the Wellcome Trust and American Foundation for Suicide Prevention, and was an expert adviser to the WHO consultation on cost-effectiveness of suicide prevention interventions, including pesticide regulation, and provided technical assistance for the development and publication of *Suicide prevention: A resource guide for pesticide registrars and regulators*. All other authors declare no competing interests.

## Supplementary Materials

Sinhala translation of the abstract

Tamil translation of the abstract
