# Supplementary material for: Case fatality of agricultural pesticides after self-poisoning in Sri Lanka: a prospective cohort study
Source: Lancet Glob Health. 2021 Apr 23;9(6):e854–62. doi: 10.1016/S2214-109X(21)00086-3 (PMC8131203; doi:10.1016/S2214-109X(21)00086-3)
Supplement: Sinhala translation of the abstract [file mmc1.pdf]

THE LANCET
Global Health

Supplementary appendix 1

This translation in Sinhala was submitted by the authors and we reproduce it as supplied. It has not been peer reviewed. The Lancet's editorial processes have only been applied to the original in English, which should serve as reference for this manuscript.

වමම සිංහල භාෂා පරිවර්තනය කතුවරුන්ත විසින්ත වයාමුකරන ලද අතර අපි එය සපයා ඇති පරිදි ඉදිරිපත් කරන්තවනමු. වේ සඳහා තුලයසමාවලෝචනයක් සිදුකර වනාමැත. ලැන්තවසට්හි සංස්කරණ ක්‍රියාවලීන්ත වයදී ඇත්වත් වේ සඳහා වයාමු කිරීමක් වන වමහි ඉංග්‍රීසි මුල් පිටපතටපමණි.

Supplement to: Buckley NA, Fahim M, Raubenheimer J, et al. Case fatality of agricultural pesticides after self-poisoning in Sri Lanka: a prospective cohort study. *Lancet Glob Health* 2021; published online April 23. http://dx.doi.org/10.1016/S2214-109X(21)00086-3.

ශ්‍රී ලංකාවේ කෘෂිකාර්මික පළිබෝධනාශක ශරීරගත කරගැනීම්වල මරණ අනුපාතය: පුරෝකථන සාමූහික අධ්‍යයනය

මහාචාර්ය නිකලස් ඒ. බක්ලි[1,2] MD, මොහොමඩ් ෆාහිම්[1,2,3] PhD, යාක් රෝබන්හයිමර්[1,2] PhD, මහාචාර්ය ඉන්දික බී. ගවරම්මාන[1] PhD, මහාචාර්ය මයිකල් එඩ්ල්ස්ටන්[1,4] PhD, මහාචාර්ය මයිකල් එස්. රොබර්ට්ස්[1,5,6] PhD, මහාචාර්ය ඇන්ඩෘ එච්. ඩෝසන්[1,2] FRCP.

1. දකුණු ආසියාතික සායනික විෂවේද පර්යේෂණ සහයෝගිතාව, වෛද්‍ය පීඨය, පේරාදෙණිය විශ්වවිද්‍යාලය
2. ඖෂධවේද, ජෛව-වෛද්‍ය තොරතුරු තාක්ෂණ හා ඩිජිටල් සෞඛ්‍ය, වෛද්‍ය හා සෞඛ්‍ය පීඨය, සිඩ්නි විශ්වවිද්‍යාලය, ඕස්ට්‍රේලියාව
3. ඖෂධවේද දෙපාර්තමේන්තුව, සම සෞඛ්‍ය විද්‍යා පීඨය, පේරාදෙණිය විශ්වවිද්‍යාලය
4. පළිබෝධනාශකමගින් දිවිනසාගැනීම් වැලැක්වීම සඳහා මධ්‍යස්ථානය, සහ හෘද විද්‍යාව සඳහා ඖෂධවේද, විෂවේද සහ චිකිත්සා මධ්‍යස්ථානය, එඩින්බරෝ විශ්වවිද්‍යාලය, එක්සත් රාජධානිය
5. පරිවර්තන සෞඛ්‍ය පර්යේෂණ සඳහා වන බැසිල් හෙට්සෙල් ආයතනය, දකුණු ඕස්ට්‍රේලියා විශ්වවිද්‍යාල සායනික සහ සෞඛ්‍ය විද්‍යා ආයතනය, දකුණු ඕස්ට්‍රේලියා විශ්වවිද්‍යාලය, ඇඩිලේඩ්.
6. ඩයමන්ටිනා ආයතනය, ක්වීන්ස්ලන්ඩ් විශ්වවිද්‍යාලය, බ්‍රිස්බේන්, ඕස්ට්‍රේලියාව

විමසීම්: නිකලස් බක්ලි - nicholas.buckley@sydney.edu.au
සායනික ඖෂධවේද මහාචාර්ය
සෞඛ්‍ය හා වෛද්‍ය පීඨය (K06)
සිඩ්නි විශ්වවිද්‍යාලය, නිව්සවුත්වේල්ස් 2006

සංක්ෂේපය

පසුබිම: පළිබෝධනාශක විෂ ශරීරගත කරගැනීම ලොව සුලබතම සියදිවි හානිකරගැනීමේ ක්‍රමයක් වුවත් හානිකර රසායන සහිත පළිබෝධනාශක වඩාත් ක්‍රමානුකූලව නියාමනය මගින් එය වළක්වාගත හැක. අප විසින් මෙම අධ්‍යනය සඳහා විවිධ පළිබෝධනාශකවල මරණ අනුපාතය සංසන්දනය කිරීම සහ කාලයත් සමඟ ශ්‍රී ලංකාවේ එම මරණ අනුපාතය වෙනස් වන ආකාරය අධ්‍යනය කිරීම අරමුණු කරගන්නා ලදී.

ක්‍රමවේදය: ශ්‍රී ලංකාවේ රෝහල් නවයක් ආශ්‍රිතව කෘෂි පළිබෝධනාශක විෂ ශරීරගතකරගැනීම් බවට හඳුනාගත් හෝ එසේ සැක කෙරෙන රෝගීන් නියැදියක මරණ අනුපාතය අප විසින් අධ්‍යයනය කරන ලදි. අධ්‍යයන කාලසීමාව තුල දකුණු ආසියාතික සායනික විෂවේද පර්යේෂණ සහයෝගිතාව හා සම්බන්ධ රෝහලකට පැමිණි අවුරුදු 11 ට වැඩි සියලුම රෝගීන් අප විසින් මේ සඳහා ඇතුළත් කරගන්න ලදී. රෝගීන් ඇතුලත් කර ගැනීම සායනික පර්යේෂණ සහායකයින් විසින් සිදු කරන ලද අතර ඔවුන් විසින් රෝගීන් නිරන්තරයෙන් පරීක්ෂාවට ලක් කරන ලදී. භාවිත කරන ලද පළිබෝධනාශක විෂ හඳුනාගැනීම, රුධිර විශ්ලේෂණය සහ රෝග ඉතිහාසය මත හෝ අසුරණය හඳුනාගැනීම මත පදනම් විය.

සොයාගැනීම්: 2002 මාර්තු 31 සිට 2019 දෙසැම්බර් 31 අතර කාලය තුළ පළිබෝධනාශක විෂ ශරීරගතකරගැනීම් බවට හඳුනාගත් රෝගීන් 34902 දෙනකුගේ නියැදියක මරණ අනුපාතය අප විසින් අධ්‍යයනය කරන ලදි. මෙම නියැදියේ මධ්‍යනාය වයස 29ක් (IQR 21-40) වූ අතර ඉන් 23,060ක් (66.1%) පුරුෂයන් විය. රෝගීන් 2,299 (6.6%) ක් සඳහා විෂ වීම මාරාන්තික විය. මරණ අනුපාතය 0.0% (බොහෝ පළිබෝධනාශක සඳහා) සිට 41.8% (පැරකොට්) අතර විශාල පරාසයක පැවතිණි. 2008 සිට 2011 දක්වා කාලය තුළ ප්‍රබලතම විෂ කාරක රසායන තුන (පැරකොට්, ඩයිමෙතොඒට්, ෆෙන්තියන්) තහනම් කරන ලදී. 2013 සිට වැඩිම මරණ අනුපාතයකට හේතු වූ විෂ කාරක රසායන පහ ප්‍රොෆෙනොෆොස්, ප්‍රොපානිල්, ෆෙනෝබියුකාර්බ්, කාබොසල්ෆාන්, ක්විනල්ෆොස් විය. මේ මගින් 7.2-8.6% අතර මරණ අනුපාතයක් පැවතිණි. සමස්තයක් ලෙස පළිබෝධනාශක විෂවීම් වල මරණ අනුපාතයේ විශාල පහත වැටීමක් සිදුවිය (2002-06 සඳහා 10.5% සිට 2013-19 සඳහා 3.7%). පළිබෝධනාශක සඳහා පැනවුණු තහනම් මීට බොහෝ දුරට හේතු විය. තහනම් කිරීමට ලක් නොවූ පළිබෝධනාශක වලද මරණ අනුපාතයේ යම් පහත වැටීමක් දැක ගත හැකි විය.

අර්ථකථනය: තහනම් කිරීමට ලක් නොවූ පළිබෝධනාශකවල මරණ අනුපාතයේ පහත වැටීම රෝග ප්‍රතිකාර කිරීමේ ඇතිවූ දියුණුවක් ලෙස හැඳින්විය හැක. කෘෂි විෂ වල හානිකරතාව වර්ගීකරණය හා නියාමනය සිදු කිරීමේදී හැකිතාක් දුරට මානව දත්ත පමණක් භාවිතා කල යුතුය. පළිබෝධනාශක ලියාපදිංචිය සඳහා මරණ අනුපාතය 5% ට අඩු පළිබෝධනාශක පමණක් තෝරා ගැනීම මඟින් බොහෝ මරණ වළක්වා ගැනීමටත් ඒ මඟින් ගෝලීය සියදිවි නසා ගැනීමේ අනුපාතයට සැලකිය යුතු බලපෑමක් ඇති කිරීමටත් හැක.

මූල්‍ය ප්‍රතිපාදන: Wellcome Trust අරමුදල (GR063560MA, GR071669MA) සහ ජාතික සෞඛ්‍ය හා වෛද්‍ය පර්යේෂණ කවුන්සිලය, ඕස්ට්‍රේලියාව (268053, 1030069, 1055176).
