# Supplementary material for: Case fatality of agricultural pesticides after self-poisoning in Sri Lanka: a prospective cohort study
Source: Lancet Glob Health. 2021 Apr 23;9(6):e854–62. doi: 10.1016/S2214-109X(21)00086-3 (PMC8131203; doi:10.1016/S2214-109X(21)00086-3)
Supplement: Tamil translation of the abstract [file mmc2.pdf]

THE LANCET
Global Health

## Supplementary appendix 2

This translation in Tamil was submitted by the authors and we reproduce it as supplied. It has not been peer reviewed. The Lancet's editorial processes have only been applied to the original in English, which should serve as reference for this manuscript.

தமிழில் இந்த மொழிபெயர்ப்பு ஆசிரியர்களால் சமர்ப்பிக்கப்பட்டது, நாங்கள் அதை வழங்கியபடி மீண்டும் உருவாக்குகிறோம்.இது மதிப்பாய்வு செய்யப்படவில்லை. லான்செட்டின் தலையங்க செயல்முறைகள் ஆங்கிலத்தில் அசலுக்கு மட்டுமே பயன்படுத்தப்பட்டுள்ளன, இது இந்த கையெழுத்துப் பிரதிக்கான குறிப்பாக செயல்பட வேண்டும்.

Supplement to: Buckley NA, Fahim M, Raubenheimer J, et al. Case fatality of agricultural pesticides after self-poisoning in Sri Lanka: a prospective cohort study. *Lancet Glob Health* 2021; published online April 23. http://dx.doi.org/10.1016/S2214-109X(21)00086-3.

**இலங்கையில் விவசாய பூச்சிக்கொல்லிகளை கொண்டு சுயவிஷம் உட்கொள்ளுதலுக்கு பிறகு இறப்பு குறித்து ஒரு வருங்கால அடிப்படையில் ஒருங்கிணைந்த ஆய்வு.**

பேராசிரியர் நிக்கோலஸ் ஏ பக்லி (எம்.டி), மொஹமட் பாஹிம் (பி.எச்.டி), ஜாக் ரூபென்ஹைமர் (பி.எச்.டி), பேராசிரியர் இந்திகா பி கவாரம்மனா (பி.எச்.டி), பேராசிரியர். மைக்கேல் எட்ல்ஸ்டன் (பி.எச்.டி), பேராசிரியர். மைக்கேல் எஸ் ராபர்ட்ஸ் (பிஎச்.டி), பேராசிரியர். ஆண்ட்ரூ எச் டாசன் (எஃப்.ஆர்.சி.பி).

1. தெற்காசிய மருத்துவ நச்சுயியல் ஆராய்ச்சி ஒத்துழைப்பு, மருத்துவ பேரவை, பெரடேனியா பல்கலைக்கழகம்
2. மருந்தியல், பயோமெடிக்கல் தகவல் மற்றும் டிஜிட்டல் உடல்நலம், மருத்துவம் மற்றும் சுகாதார பேரவை, சிட்னி பல்கலைக்கழகம், ஆஸ்திரேலியா
3. இணை சுகாதார அறிவியல் பேரவை, மருந்தியல் துறை, பெரடேனியா பல்கலைக்கழகம், இலங்கை
4. பூச்சிக்கொல்லி தற்கொலை தடுப்பு மையம், மற்றும் மருந்தியல், நச்சுயியல் மற்றும் சிகிச்சை முறைகள், இருதய அறிவியல் மையம், எடின்பர்க் பல்கலைக்கழகம், இங்கிலாந்து.
5. மாற்றியமைப்பு சுகாதார ஆராய்ச்சிக்கான பசில் ஹெட்செல் நிறுவனம், யுனிசா மருத்துவ மற்றும் சுகாதார அறிவியல், தெற்கு ஆஸ்திரேலியா பல்கலைக்கழகம், அடிலெய்ட்
6. டயமண்டினா நிறுவனம், குயின்ஸ்லாந்து பல்கலைக்கழகம், பிரிஸ்பேன், ஆஸ்திரேலியா

மின்னஞ்சல்/அஞ்சல் தொடர்பு:நிக்கோலஸ் பக்கலே nicholas.buckley@sydney.edu.au
மருத்துவ மருந்தியல் பேராசிரியர்,
மருத்துவம் மற்றும் சுகாதார பேரவை (K06),
சிட்னி பல்கலைக்கழகம், NSW 2006.

**இலங்கையில் விவசாய பூச்சிக்கொல்லிகளை கொண்டு சுய-விஷம் உட்கொள்ளுதலுக்கு பிறகு இறப்பு குறித்து: ஒரு வருங்கால அடிப்படையில் ஒருங்கிணைந்த ஆய்வு.**

*பேராசிரியர் நிக்கோலஸ் ஏ பக்லி (எம்.டி), மொஹமட் பாஹிம் (பி.எச்.டி), ஜாக் ரூபென்ஹைமர் (பி.எச்.டி) பேராசிரியர் இந்திகா பி கவாரம்மனா (பி.எச்.டி), பேராசிரியர். மைக்கேல் எட்ல்ஸ்டன் (பி.எச்.டி), பேராசிரியர். மைக்கேல் எஸ் ராபர்ட்ஸ் (பிஎச்.டி), பேராசிரியர். ஆண்ட்ரூ எச் டாசன் (எஃப்.ஆர்.சி.பி).*

**பிண்ணனி**: பூச்சிக்கொல்லி விஷத்தை அருந்துதல் என்பது உலகிலேயே தற்கொலைக்காக மிகவும் பரவலாகப் பயன்படுத்தப்படும் ஒரு வழிமுறையாகும். எனினும் ஆபத்தான பூச்சிக்கொல்லிகளை சீரான முறையில் ஒழுங்குபடுத்துவதன் மூலம் இவ்வாறான உயிரிழப்புகளை தவிர்த்துக்கொள்ளலாம். அருந்தப்பட்ட பூச்சுக்கொல்லிகளின் வலிமையை ஒப்பிட்டுப் பார்ப்பது, மனித மற்றும் விலங்கு நச்சுத்தன்மைக்கு இடையிலான மாறுபாடு குறித்த ஆதாரங்களைத் தேடுவது மற்றும் காலப்போக்கில் இலங்கையில் தனிப்பட்ட பூச்சிக்கொல்லிகளை உட்கொள்வதன் மூலம் ஏற்படும் இறப்புகளில் மாற்றத்தை கண்டறிதல் போன்றவற்றை ஆராய்வதே இவ்வாய்வின் நோக்கமாகும்

**முறைகள்:** நாங்கள் ஒருங்கிணைந்த ஆய்வின் மூலம் கிராமப்புறங்களுக்களில் சேவை செய்யும் ஒன்பது மருத்துவமனைகளுக்கு அனுமதிக்கப்பட்ட பூச்சிக்கொல்லி அருந்தி மரணிப்பவர்களின் எண்ணிக்கையை கண்காணித்தோம். ஆய்வு காலத்தில் மருத்துவமனை தேற்காசிய நச்சுயியல் ஆய்வு நிலையத்திற்கு வருகை தந்த அணைத்து நோயாளர்களையும் (>11 வருடம்) ஆய்வில் இணைத்துக்கொண்டோம். மருத்துவ ஆராய்ச்சி உதவியாளர்களால் நோயாளர்களை கண்காணித்து, தொடர் மதிப்பாய்வுக்கு உட்படுத்தப்படுவதன் மூலம், உட்கொண்ட பூச்சிக்கொல்லிகளை அடையாளம் காண்பதற்கு வரலாறு அல்லது அடையாளம் காணப்பட்ட பூச்சிக்கொல்லி கொள்கலன்கள் மற்றும் குறுதிப்பகுப்பாய்வு தகவல்கள் கொண்டு கண்டறிந்தோம்.

**கண்டுபிடிப்புகள்:** மார்ச் 31, 2002 முதல் டிசம்பர் 31, 2019 வரை 34 902 நோயாளிகள் (சராசரி வயது 29 வருடம் [IQR 21-40]; 23 060 [66 · 1%] ஆண்) சாத்தியமான அல்லது உறுதிப்படுத்தப்பட்ட பூச்சிக்கொல்லிகளால் சுயவிஷப்படுத்தப்பட்டு அனுமதிக்கப்பட்டனர், உட்கொள்ளப்பட்ட 23139 குறிப்பிட்ட வகையான பூச்சிக்கொல்லிகளை நாங்கள் அடையாளம் கண்டோம். விஷம் அருந்திய நோயாளர்களில் 2999 (6.6%) அபாயகரமான நிலைமையில் உள்ளதோடு இறப்பு விகிதம் 0% (பல பூச்சுக்கொல்லிகள்) முதல் 41.8% (பராகுவட்) வரை பெருமளவில் மாறுபட்டன. மூன்று பிரதான நச்சு வகைகள் (அதாவது, பராகுவட், டைமெத்தோயேட் மற்றும் பெந்தியோன்) ஆகியன 2008 முதல் 2011 வரையான காலப்பகுதியில் தடைசெய்யப்பட்டன. 2013 முதல், பாரிய மரண அச்சுறுத்தலை ஏற்படுத்திய ஐந்து நச்சுவகைகள்; (புரோபெனாபோஸ், புரோபெனில், பெனோபுகார்ப், கார்போசுல்பான், குயினால்போஸ்) ஆகியன 7-9% பாதிப்புகளை ஏற்படுத்தியுள்ளன. பூச்சுக்கொல்லிகள் அருந்தி விஷப்படுத்தப்படுவதினால் ஏற்பட்ட ஒட்டுமொத்த இறப்பின் எண்ணிக்கையில் நிலையான வீழ்ச்சியை காண முடிந்ததோடு (2002–06 ஆம் ஆண்டில் 10.5% முதல் 2013-19 வரை 3.7% வரை), பெரும்பாலும் இப்பூச்சிக்கொல்லிகளின்

தடையே இதற்கான காரணமாகும். தடை செய்யப்படாத பூச்சிக்கொல்லிகளினால் ஏற்பபடும் இறப்பில் ஒரு சாதாரண வீழிச்சியையும் காண முடிந்தது.

**அர்த்தவிளக்கப்படுத்தல்:** தரமான சிகிச்சை முறைகள் உள்வாங்கப்படுவதினால், கட்டுப்படுத்தப்படாத பூச்சுக்கொல்லிகளால் ஏற்படும் உயிரிழப்பு கணிசமாக குறைந்துள்ளது. பூச்சிக்கொல்லிகளால் கடுமையாக விஷமாக்கப்படுத்தல் சம்பந்தமான மனித தரவுகள், ஆபத்தான பூச்சிக்கொல்லிகளை வகைபடுத்தல் மற்றும் சீரான முறைகளை ஒழுங்கு படுத்தல் போன்றவற்றை வலியுறுத்துகின்றது. மேலும் இந்த ஆய்வின் இன்னுமொரு கருத்தானது பூச்சிக்கொல்லிகளின் பதிவுகளின்போது 5% இற்கும் குறைவான மரணங்களை ஏற்படுத்தும் பூச்சிக்கொல்லிகளை பதிவு செய்வதின் மூலம் மேலும் பல மரணங்களை தவிர்த்துக்கொள்ள முடிவதோடு, உலக தற்கொலை வீததில் இது பாரிய மாற்றத்தை ஏற்படுத்தும்.

**நிதியுதவி:** த வெல்கம் டிரஸ்ட் (T Wellcome Trust) (GR063560MA, GR071669MA) தேசிய சுகாதாரம் மற்றும் மருத்துவ ஆய்வு ஆணைக்குழு, அவுஸ்திரேலியா (268053, 1030069, 1055176).
