# Supplementary material for: Case fatality of agricultural pesticides after self-poisoning in Sri Lanka: a prospective cohort study
Source: Lancet Glob Health. 2021 Apr 23;9(6):e854–62. doi: 10.1016/S2214-109X(21)00086-3 (PMC8131203; doi:10.1016/S2214-109X(21)00086-3)
Supplement: Supplementary appendix 3 [file mmc3.pdf]

THE LANCET
Global Health

Supplementary appendix 3

This appendix formed part of the original submission and has been peer reviewed. We post it as supplied by the authors.

Supplement to: Buckley NA, Fahim M, Raubenheimer J, et al. Case fatality of agricultural pesticides after self-poisoning in Sri Lanka: a prospective cohort study. *Lancet Glob Health* 2021; published online April 23. http://dx.doi.org/10.1016/S2214-109X(21)00086-3.

Supplementary Tables 1 to 5 for Buckley NA et al. **Case fatality of agricultural pesticides after self-poisoning in Sri Lanka: a prospective cohort study. Lancet Global Health 2021.**

Supplementary Table 1 – Dates of initiation of phased in partial restrictions and subsequent importation bans and legislative restrictions on pesticides in Sri Lanka.

**Pesticide**	**Partial restriction/ Phase out period starts**	**Effective Restrictions**	**Legislative ban**
Most POPs		1970-95	2001
**Class I pesticides**		1984-95	2001
**Endosulfan**		1998	2001
**Paraquat**	2008	2012	2014
**Dimethoate**	2008	2011	2014
**Fenthion**	2008	2011	2014
Cyromazine	?	2011	2014
Alachlor	?	2012	2014
Glyphosate	2014 †	2015	* 2015
Carbofuran	2013	2015	2016
Carbaryl	2013	2015	2016
Chlorpyrifos	2013	2015	2016
Propanil	2014 †		-

* In 2018 reregistered with tight restrictions

† Regional restriction

Bolded agents restricted to reduce risks from acute poisoning, other restrictions for environmental or chronic toxicity.

Internet sources [11,21]: https://www.doa.gov.lk/SCPPC/images/ROP/Tabel.pdf

https://www.parliament.lk/uploads/documents/paperspresented/performance-report-department-of-agriculture-2015.pdf

Supplementary Table 2. Characteristics of cohort, overall and in three different time periods.

	Overall		2002-2006		2007-2012		2013-2019	
	**N**	**Median (IQR) or N (%)**	**N**	**Median (IQR) or N (%)**	**N**	**Median (IQR) or N (%)**	**N**	**Median (IQR) or N (%)**
**N transferred**	34,902	23,245 (66.6%)	7,741	5230 (67.6%)	15,958	10,900 (68.3%)	11,203	7,115 (63.5%)
**Deaths**	34,902	2,299 (6.6%)	7,741	815 (10.5%)	15,958	1,073 (6.7%)	11,203	411 (3.67%)
**Sex**	34,900		7,741		15,956		11,203	
**Female**		11,840 (33.9%)		2,532 (32.7%)		5,604 (35.1%)		3,704 (33.1%)
**Male**		23,060 (66.1%)		5,209 (67.3%)		10,352 (64.9%)		7,499 (66.9%)
**Age**	34,898	29 (21 - 40)	7,741	28 (21 - 39)	15,955	28 (21 - 40)	11,202	30 (22 - 43)
**Time post ingestion (mins) ***	32,114	225 (130 - 405)	7,527	261 (171 - 422)	14,456	262 (150 - 520)	10,131	160 (90 - 260)
**Ingested multiple pesticides**	34,902	455 (1.3%)	7,741	166 (2.1%)	15,958	206 (1.3%)	11,203	83 (0.7%)
**Cohort ingestions**	35,628		7,999		16,221		11,408	
**Known pesticides**		23,139 (64.9%)		6,158 (77.0%)		10,700 (66.0%)		6,281 (55.1%)
**'Unknown pesticides'**		6,643 (18.6%)		1,198 (15.0%)		2,838 (17.5%)		2,607 (22.9%)
**'Unknown substances'**		5,846 (16.4%)		643 (8.0%)		2,683 (16.5%)		2,520 (22.1%)

* Taken from time of first recorded examination in 48% of cases where admission time was not recorded.

Supplementary Table 3 Case fatality of all pesticides and unknown substances ordered by class and then case numbers as identified in the SACTRC database

Class	Substance	N of Patients	Fatal	Case fatality	95%CI
**Organophosphorus insecticide**		10612	927	8.7%	
	Chlorpyrifos	3340	213	6.4%	(5.6 to 7.3)
	Unknown OP	2637	234	8.9%	(7.8 to 10)
	Dimethoate	1190	229	19.2%	(17 to 21.6)
	Profenofos	1161	84	7.2%	(5.8 to 8.9)
	Malathion	482	12	2.5%	(1.3 to 4.3)
	Diazinon	448	27	6.0%	(4 to 8.6)
	Fenthion	388	56	14.4%	(11.1 to 18.3)
	Phenthoate	366	25	6.8%	(4.5 to 9.9)
	Quinalphos	303	26	8.6%	(5.7 to 12.3)
	Acephate	51	1	2.0%	(0 to 10.4)
	Coumaphos	35	0	0.0%	(0 to 10)
	Methamidophos	35	3	8.6%	(1.8 to 23.1)
	Monocrotophos	29	1	3.4%	(0.1 to 17.8)
	Azamethiphos	25	0	0.0%	(0 to 13.7)
	Prothiofos	22	3	13.6%	(2.9 to 34.9)
	Pirimiphos-Methyl	20	0	0.0%	(0 to 16.8)
	Phoxim	14	1	7.1%	(0.2 to 33.9)
	Azinphos Methyl	12	1	8.3%	(0.2 to 38.5)
	Oxydemeton-Methyl	8	1	12.5%	(0.3 to 52.7)
	Dichlorvos	7	0	0.0%	(0 to 41)
	Parathion Methyl	7	5	71.4%	(29 to 96.3)
	Triazophos	5	0	0.0%	(0 to 52.2)
	Parathion	4	1	25.0%	(0.6 to 80.6)
	Carbophenothion	3	0	0.0%	(0 to 70.8)
	Chlorfenvinphos	3	0	0.0%	(0 to 70.8)
	Formothion	3	1	33.3%	(0.8 to 90.6)
	Bromophos Ethyl	2	1	50.0%	(1.3 to 98.7)
	Fenitrothion	2	0	0.0%	(0 to 84.2)
	Phosphate	2	0	0.0%	(0 to 84.2)
	Ethion	1	1	100.0%	(2.5 to 100)
	Etrimfos	1	0	0.0%	(0 to 97.5)
	Isofenphos	1	1	100.0%	(2.5 to 100)
	Phosalone	1	0	0.0%	(0 to 97.5)
	Temephos	1	0	0.0%	(0 to 97.5)
	Tetrachlorvinphos	1	0	0.0%	(0 to 97.5)

	Trichlorfon	1	0	0.0%	(0 to 97.5)
	Phosphorothioate	1	0	0.0%	(0 to 97.5)
**Carbamates**		3440	165	4.8%	
	Carbofuran	1740	37	2.1%	(1.5 to 2.9)
	Carbosulfan	1129	86	7.6%	(6.1 to 9.3)
	Fenobucarb (BPMC)	315	27	8.6%	(5.7 to 12.2)
	Unknown Carbamate	125	11	8.8%	(4.5 to 15.2)
	Propoxur	68	1	1.5%	(0 to 7.9)
	Carbaryl	30	2	6.7%	(0.8 to 22.1)
	Methomyl	26	1	3.8%	(0.1 to 19.6)
	Diazylhydrazine	4	0	0.0%	(0 to 60.2)
	Dimethylcarbamate	1	0	0.0%	(0 to 97.5)
	Isoprocarb	1	0	0.0%	(0 to 97.5)
	Mexacarbate	1	0	0.0%	(0 to 97.5)
**Pyrethroids**		1046	5	0.5%	
	Etofenprox	482	4	0.8%	(0.2 to 2.1)
	Unknown Pyrethroid	260	0	0.0%	(0 to 1.4)
	Allethrin	85	0	0.0%	(0 to 4.2)
	Flumethrin	51	0	0.0%	(0 to 7)
	Cypermethrin	42	0	0.0%	(0 to 8.4)
	Deltamethrin	32	0	0.0%	(0 to 10.9)
	Lambda-Cyhalothrin	16	0	0.0%	(0 to 20.6)
	Imiprothrin	14	0	0.0%	(0 to 23.2)
	Cyfluthrin	12	0	0.0%	(0 to 26.5)
	Fenvalerate	11	1	9.1%	(0.2 to 41.3)
	Transfluthrin	9	0	0.0%	(0 to 33.6)
	Esfenvalerate	7	0	0.0%	(0 to 41)
	Cyhalothrin	4	0	0.0%	(0 to 60.2)
	Phenothrin	4	0	0.0%	(0 to 60.2)
	Prallethrin	4	0	0.0%	(0 to 60.2)
	Pyrethrum	4	0	0.0%	(0 to 60.2)
	Pybuthrin	2	0	0.0%	(0 to 84.2)
	Resmethrin	2	0	0.0%	(0 to 84.2)
	Tetramethrin/Cyphenothrin	2	0	0.0%	(0 to 84.2)
	Cypermethrin/Imiprothrin	1	0	0.0%	(0 to 97.5)
	Cyphenothrin	1	0	0.0%	(0 to 97.5)
	Phenothrine	1	0	0.0%	(0 to 97.5)
**Other Insecticides**		1008	20	2.0%	
	Abamectin	254	4	1.6%	(0.4 to 4)
	Imidacloprid	218	8	3.7%	(1.6 to 7.1)
	Fipronil	122	0	0.0%	(0 to 3)
	Chlorfluazuron	110	2	1.8%	(0.2 to 6.4)
	Thiamethoxam	56	1	1.8%	(0 to 9.6)

	Acetamiprid	42	0	0.0%	(0 to 8.4)
	Tebufenozide	29	1	3.4%	(0.1 to 17.8)
	Unknown Neonicotinoid	18	0	0.0%	(0 to 18.5)
	Chlorantraniliprole	17	1	5.9%	(0.1 to 28.7)
	Endosulfan	15	3	20.0%	(4.3 to 48.1)
	Benzoylurea	14	0	0.0%	(0 to 23.2)
	Azadirachtin	11	0	0.0%	(0 to 28.5)
	Spinosad	10	0	0.0%	(0 to 30.8)
	Novaluron	8	0	0.0%	(0 to 36.9)
	Chromafenozide	7	0	0.0%	(0 to 41)
	Mortein	7	0	0.0%	(0 to 41)
	Amitraz	5	0	0.0%	(0 to 52.2)
	Indoxacarb	5	0	0.0%	(0 to 52.2)
	Methoxyfenozide	5	0	0.0%	(0 to 52.2)
	Phenylpyrazole	5	0	0.0%	(0 to 52.2)
	Emamectin Benzoate	4	0	0.0%	(0 to 60.2)
	Flubendiamide	4	0	0.0%	(0 to 60.2)
	Thiacloprid	4	0	0.0%	(0 to 60.2)
	Insecticide Chalk	3	0	0.0%	(0 to 70.8)
	Mosquito Coil	3	0	0.0%	(0 to 70.8)
	Thiocyclam Hydrogen Oxalate	3	0	0.0%	(0 to 70.8)
	Unknown Organochlorine	3	0	0.0%	(0 to 70.8)
	Ant Poison	2	0	0.0%	(0 to 84.2)
	Chloronicotinyl	2	0	0.0%	(0 to 84.2)
	Cyromazine	2	0	0.0%	(0 to 84.2)
	Insect Growth Regulator	2	0	0.0%	(0 to 84.2)
	Lufenuron	2	0	0.0%	(0 to 84.2)
	Tick Poison	2	0	0.0%	(0 to 84.2)
	Aluminium Phosphide	1	0	0.0%	(0 to 97.5)
	Ant Killer	1	0	0.0%	(0 to 97.5)
	Arsenic	1	0	0.0%	(0 to 97.5)
	Buprofezine	1	0	0.0%	(0 to 97.5)
	Carbavin	1	0	0.0%	(0 to 97.5)
	Clofentezine	1	0	0.0%	(0 to 97.5)
	Flufenoxuron	1	0	0.0%	(0 to 97.5)
	Methylene Blue	1	0	0.0%	(0 to 97.5)
	Mosquito Repellant	1	0	0.0%	(0 to 97.5)
	Paradichlorobenzene	1	0	0.0%	(0 to 97.5)
	Rynaxypyr	1	0	0.0%	(0 to 97.5)
	Sulfluoramide	1	0	0.0%	(0 to 97.5)
	Unknown Insect Growth Regulator	1	0	0.0%	(0 to 97.5)
	Unknown Phenylpyrazole	1	0	0.0%	(0 to 97.5)
**Herbicides**		9054	904	10.0%	

Glyphosate	3908	93	2.4%	(1.9 to 2.9)
MCPA	1646	88	5.3%	(4.3 to 6.5)
Paraquat	1477	618	41.8%	(39.3 to 44.4)
Propanil	982	81	8.2%	(6.6 to 10.1)
Bispyribac-Sodium	227	7	3.1%	(1.2 to 6.3)
Fenoxaprop-P-Ethyl	173	1	0.6%	(0 to 3.2)
Unknown Herbicide	139	2	1.4%	(0.2 to 5.1)
Pretilachlor	119	2	1.7%	(0.2 to 5.9)
Pyribenzoxim	59	1	1.7%	(0 to 9.1)
Glufosinate Ammonium	48	3	6.3%	(1.3 to 17.2)
Oxyfluorfen	34	0	0.0%	(0 to 10.3)
Clomazone	26	2	7.7%	(0.9 to 25.1)
Quinclorac	24	3	12.5%	(2.7 to 32.4)
Ethephon	22	0	0.0%	(0 to 15.4)
Pendimethalin	20	0	0.0%	(0 to 16.8)
Dithiocarbamate	19	0	0.0%	(0 to 17.6)
Thiodicarb	18	1	5.6%	(0.1 to 27.3)
Diuron	14	0	0.0%	(0 to 23.2)
Alachlor	13	1	7.7%	(0.2 to 36)
Benthiocarb	11	0	0.0%	(0 to 28.5)
Azimsulfuron	9	0	0.0%	(0 to 33.6)
Oxadiazon	9	0	0.0%	(0 to 33.6)
Bensulfuron-Methyl	6	0	0.0%	(0 to 45.9)
Ethoxysulfuron	6	0	0.0%	(0 to 45.9)
1-Naphthaleneacetic Acid	5	0	0.0%	(0 to 52.2)
2,4-Dichlorophenoxyacetic Acid	5	0	0.0%	(0 to 52.2)
Metribuzin	5	0	0.0%	(0 to 52.2)
Chlorophenoxy	4	1	25.0%	(0.6 to 80.6)
Cyhalofop-Butyl Propionate	3	0	0.0%	(0 to 70.8)
Carfentrazone-Ethyl	2	0	0.0%	(0 to 84.2)
Nitrophenolate	2	0	0.0%	(0 to 84.2)
Orthosulfamuron	2	0	0.0%	(0 to 84.2)
Penoxsulam	2	0	0.0%	(0 to 84.2)
Pyrazosulfuron-Ethyl	2	0	0.0%	(0 to 84.2)
Tiafenacil	2	0	0.0%	(0 to 84.2)
3,4-Dichloropropionanilide	1	0	0.0%	(0 to 97.5)
Bromoxynil	1	0	0.0%	(0 to 97.5)
Chlorimuron Ethyl	1	0	0.0%	(0 to 97.5)
Chloroacetamide	1	0	0.0%	(0 to 97.5)
Dicholorophenol	1	0	0.0%	(0 to 97.5)
Metamifop	1	0	0.0%	(0 to 97.5)
Methyl 3,4 Dichloro Carbanilate	1	0	0.0%	(0 to 97.5)
Propachlor	1	0	0.0%	(0 to 97.5)

	Sethoxydim	1	0	0.0%	(0 to 97.5)
	Unknown Nitrophenyl Ether Herbicide	1	0	0.0%	(0 to 97.5)
	Unknown Phenol	1	0	0.0%	(0 to 97.5)
**Fungicides**		352	6	1.7%	
	Chlorothalonil	65	0	0.0%	(0 to 5.5)
	Mancozeb	36	0	0.0%	(0 to 9.7)
	Propineb	32	0	0.0%	(0 to 10.9)
	Tebuconazole	32	0	0.0%	(0 to 10.9)
	Hexaconazole	28	1	3.6%	(0.1 to 18.3)
	Unknown Fungicide	25	0	0.0%	(0 to 13.7)
	Copper Oxide	23	0	0.0%	(0 to 14.8)
	Carbendazim	19	0	0.0%	(0 to 17.6)
	Edifenphos	19	2	10.5%	(1.3 to 33.1)
	Thiram	9	1	11.1%	(0.3 to 48.2)
	Isoprothiolane	8	0	0.0%	(0 to 36.9)
	Antracol	7	0	0.0%	(0 to 41)
	Copper Sulphate	7	0	0.0%	(0 to 41)
	Propiconazole	7	0	0.0%	(0 to 41)
	Pyraclostrobin	4	1	25.0%	(0.6 to 80.6)
	Benzimidazole	3	0	0.0%	(0 to 70.8)
	Thiocarbamate	3	0	0.0%	(0 to 70.8)
	Thiophanate-Methyl	3	0	0.0%	(0 to 70.8)
	Captan	2	0	0.0%	(0 to 84.2)
	Carboxin	2	0	0.0%	(0 to 84.2)
	Coco Dimethyl Benzyl Ammonium Chloride	2	0	0.0%	(0 to 84.2)
	Fluazinam	2	0	0.0%	(0 to 84.2)
	Maneb	2	0	0.0%	(0 to 84.2)
	Propamocarb	2	1	50.0%	(1.3 to 98.7)
	Binapacryl	1	0	0.0%	(0 to 97.5)
	Bitertanol	1	0	0.0%	(0 to 97.5)
	Cosavet	1	0	0.0%	(0 to 97.5)
	Difenoconazole	1	0	0.0%	(0 to 97.5)
	Ethylene Bis-Dithiocarbamate	1	0	0.0%	(0 to 97.5)
	Hexachlorobenzene	1	0	0.0%	(0 to 97.5)
	Metiram	1	0	0.0%	(0 to 97.5)
	Picoxystrobin	1	0	0.0%	(0 to 97.5)
	Pyrazophos	1	0	0.0%	(0 to 97.5)
	Thiocyanomethyl	1	0	0.0%	(0 to 97.5)
**Rodenticides**		859	12	1.4%	
	Zinc Phosphide	635	12	1.9%	(1 to 3.3)
	Bromadiolone	98	0	0.0%	(0 to 3.7)

Coumarin	39	0	0.0%	(0 to 9)
Brodifacoum	36	0	0.0%	(0 to 9.7)
Unknown Rodenticide	29	0	0.0%	(0 to 11.9)
Ethanethiol	10	0	0.0%	(0 to 30.8)
Coumatetralyl	7	0	0.0%	(0 to 41)
Rat Poison	2	0	0.0%	(0 to 84.2)
Thallium Sulfate	2	0	0.0%	(0 to 84.2)
Difenacoum	1	0	0.0%	(0 to 97.5)
**Other Pesticides**	10	1	10.0%	
Metam Sodium	4	1	25.0%	(0.6 to 80.6)
Metaldehyde	3	0	0.0%	(0 to 70.8)
Calcium Metaphosphate	1	0	0.0%	(0 to 97.5)
Nitroguaiacol	1	0	0.0%	(0 to 97.5)
Unknown Aldehyde	1	0	0.0%	(0 to 97.5)
**'Unknown Pesticide'**	3402	157	4.6%	
**Unidentified substances**	5845	216	3.7%	

Supplementary Table 4. Effect of adjustment for age, sex, and year (with Multivariable logistic regression) on the relative odds of death with the nine most common pesticides

		Unadjusted Odds Ratio (95% CI)		Adjusted Odds Ratio (95% CI)	
**Chlorpyrifos**		[Ref]		[Ref]	
**Carbofuran**		0.32	(0.22 to 0.45)	0.38	(0.26 to 0.54)
**Carbosulfan**		1.21	(0.93 to 1.57)	1.85	(1.4 to 2.43)
**Dimethoate**		3.50	(2.86 to 4.27)	3.30	(2.68 to 4.06)
**Glyphosate**		0.36	(0.28 to 0.46)	0.55	(0.42 to 0.71)
**MCPA**		0.83	(0.64 to 1.07)	1.03	(0.79 to 1.34)
**Paraquat**		10.56	(8.89 to 12.56)	16.58	(13.71 to 20.06)
**Profenofos**		1.15	(0.88 to 1.49)	1.43	(1.08 to 1.89)
**Propanil**		1.32	(1.01 to 1.72)	1.66	(1.26 to 2.19)
**Unidentified substances**		0.56	(0.46 to 0.68)	0.87	(0.71 to 1.07)
**Unknown Organophosphates**		1.43	(1.18 to 1.73)	1.72	(1.4 to 2.12)
**Unknown Pesticides**		0.71	(0.57 to 0.88)	0.94	(0.75 to 1.17)
**Female**				0.68	(0.6 to 0.76)
**Age (per year)**				1.06	(1.05 to 1.06)
**Year**	2002			[Ref]	
	2003			0.95	(0.7 to 1.3)
	2004			1.03	(0.78 to 1.36)
	2005			0.74	(0.55 to 0.98)
	2006			0.71	(0.53 to 0.94)
	2007			0.77	(0.59 to 1)
	2008			0.52	(0.39 to 0.68)
	2009			0.43	(0.32 to 0.59)
	2010			0.48	(0.36 to 0.65)
	2011			0.34	(0.25 to 0.46)
	2012			0.26	(0.19 to 0.35)
	2013			0.24	(0.18 to 0.33)
	2014			0.26	(0.18 to 0.36)
	2015			0.34	(0.24 to 0.47)
	2016			0.40	(0.28 to 0.57)
	2017			0.26	(0.16 to 0.43)
	2018			0.29	(0.15 to 0.55)
	2019			0.33	(0.15 to 0.74)

Supplementary Table 5. Logistic regression of risk of death in the cohort comparing the effects of bans versus other factors changing over time.

Note: The percentage of patients with a known pesticide ingestion taking paraquat, dimethoate or fenthion that year is the first factor – and is equivalent to a halving of the risk of death for every 13% reduction in the proportion of pesticide poisonings taking these three highly toxic agents.

Variable		Adjusted Odds Ratio (95% CI)	
**Paraquat/Dimethoate/Fenthion (per %)**		1.055	(1.053 to 1.057)
**Age (per year)**		1.060	(1.056 to 1.063)
**Female**		0.608	(0.534 to 0.692)
**Site**	Anuradhapura	[Ref]	
	Galle	0.961	(0.791 to 1.169)
	Kurunegala	1.393	(1.152 to 1.684)
	Peradeniya	1.156	(0.968 to 1.382)
	Polonnaruwa	1.362	(1.174 to 1.581)
	Smaller Centres	1.2	(0.995 to 1.448)
**Study Year**	2002	[Ref]	
	2003	1.004	(0.734 to 1.374)
	2004	1.205	(0.908 to 1.599)
	2005	0.892	(0.664 to 1.197)
	2006	1.005	(0.753 to 1.341)
	2007	0.942	(0.714 to 1.242)
	2008	0.722	(0.543 to 0.961)
	2009	0.728	(0.532 to 0.995)
	2010	0.774	(0.561 to 1.067)
	2011	0.643	(0.472 to 0.876)
	2012	0.555	(0.403 to 0.765)
	2013	0.596	(0.434 to 0.819)
	2014	0.551	(0.395 to 0.769)
	2015	0.809	(0.576 to 1.137)
	2016	0.938	(0.664 to 1.325)
	2017	0.54	(0.338 to 0.865)
	2018	0.697	(0.368 to 1.318)
	2019	0.596	(0.282 to 1.262)
